# Supplementary material for: Reliability, Validity, and Feasibility of the Frail-VIG Index
Source: Int J Environ Res Public Health. 2021 May 13;18(10):5187. doi: 10.3390/ijerph18105187 (PMC8153117; doi:10.3390/ijerph18105187)
Supplement: Supplementary file 1 [file ijerph-18-05187-s001.zip › ijerph-1187005-supplementary.pdf]

Reliability, Validity and Feasibility of the Frail-VIG Index

Supplementary File 1

Table S1. Missing variables in the different follow-up cut-offs.

		N	%
Baseline	No miss	11594	0.00
	Miss	0	
Admission	No miss	11594	0.00
	Miss	0	
Discharge	No miss	8592	0.12
	Miss	10	
Month 1	No miss	7061	0.01
	Miss	1	
Month 6	No miss	3908	1.31
	Miss	52	
Month 9	No miss	4247	1.51
	Miss	65	
Month 12	No miss	2840	1.46
	Miss	42	
TOTAL	No miss	49836	0.34
	Miss	170	
